# Methotrexate and triamcinolone as a nonsurgical management for polymethyl methacrylate-induced granulomatous reaction: A case report

**DOI:** 10.1016/j.jdcr.2026.01.040

**Published:** 2026-01-31

**Authors:** Rodrigo do Carmo Silva, Beda Mühleisen, Roberta Vasconcelos-Berg, Alexander A. Navarini, Paula Valentina Bonavia

**Affiliations:** a Margarethenklinik Ästhetik und Dermatologie, University Hospital of Basel, Basel, Switzerland; b Department of Dermatology, University Hospital of Basel, Basel, Switzerland

**Keywords:** delayed reaction, fillers complication, foreign body granuloma, granulomatous reaction, methotrexate, PMMA, polymethyl methacrylate, treatment

## Introduction

The number of aesthetic procedures has increased substantially over the past decade, particularly in the last 5 years following the COVID-19 pandemic, with an overall growth of about 40% compared with previous years. Worldwide, aesthetic procedures have shown a steady rise, with 34.9 million surgical and nonsurgical interventions performed in 2023 by board-certified plastic surgeons, representing a 40% global increase over the last 4 years.^1^

Polymethyl methacrylate (PMMA) is a nonbiodegradable acrylic material used as a permanent filler. It consists of microspheres suspended in a colloidal gel vehicle. Despite its low cost and promise of long-lasting results, PMMA carries a risk of acute and delayed complications that are often difficult to manage.^2^, ^3^, ^4^

Late-onset adverse reactions have been reported up to 24 months after treatment, with exceptional cases occurring more than a decade after implantation.^2^^,^^5^ These reactions typically present as erythema, nodules, and edema of variable severity, frequently resulting in significant aesthetic deformity.^2^, ^3^, ^4^

Because standardized management protocols are lacking, these reactions remain a therapeutic challenge for dermatologists and plastic surgeons. Surgical removal of the injected PMMA is considered the most effective treatment;^2^ however, nonsurgical approaches have also been explored with promising results.^3^, ^4^, ^5^

We present a case of delayed inflammatory reaction associated with PMMA filler successfully managed with a combination therapy of oral methotrexate and triamcinolone injections.

## Case report

**Fig 1:**
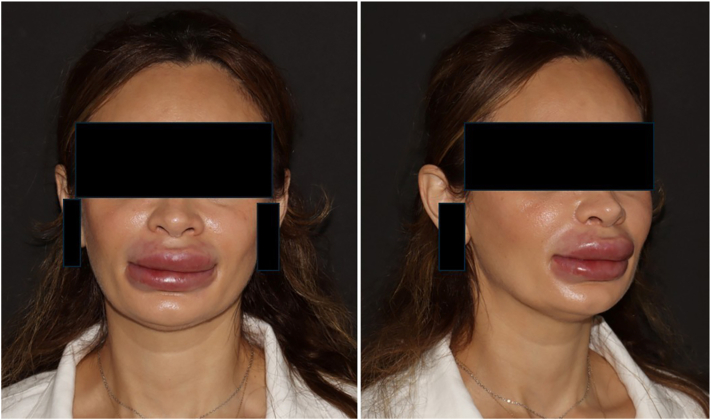
Patient before initiation of oral methotrexate therapy, presenting with marked edema of both lips and irregular livedo throughout the perioral area.

**Fig 2:**
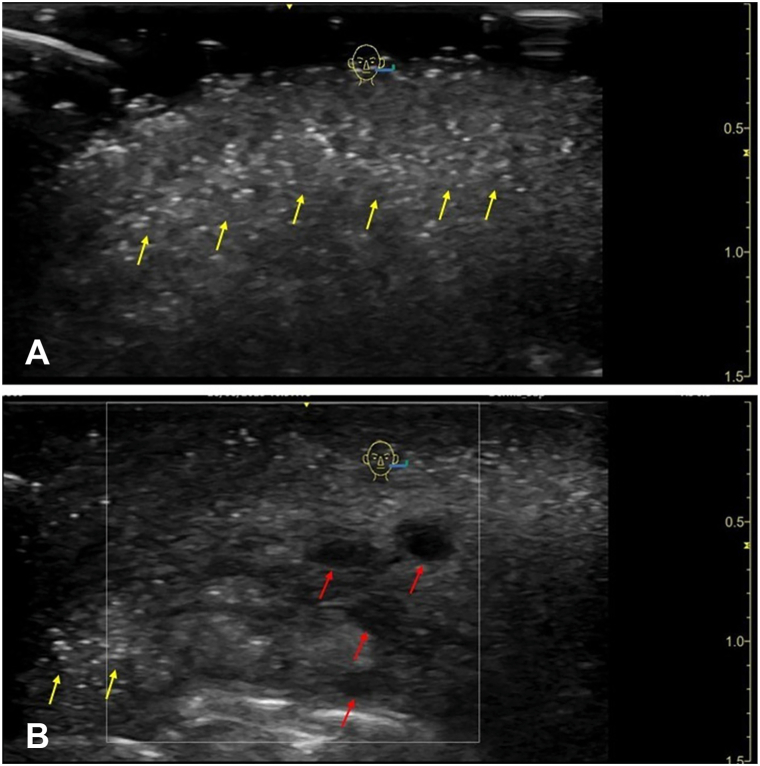
B-mode ultrasonography using a linear L8-12 transducer demonstrating: **A,** multiple bright hyperechoic foci with scattered comet-tail artifacts (*yellow arrows*) and mild posterior acoustic shadowing in the subcutaneous plane of the lips and perioral region, consistent with PMMA filler deposits; and **(B)** several anechoic pseudocysts (*red arrows*), consistent with the typical ultrasonographic appearance of hyaluronic acid fillers, and some comet-tail artifacts (*yellow arrows*) on the left. *PMMA*, Polymethyl methacrylate.

A 40-year-old woman presented to the dermatology department of the University Hospital Basel, Switzerland with nodules, erythema, and edema in the perioral region, especially the upper lip, which had started 3 days earlier (Fig 1). Ultrasonography of the lips and perioral region revealed exogenous material within the subcutaneous plane, displaying 2 distinct patterns: (a) Hyper-echogenic foci, possibly consistent with PMMA polymer deposits. (b) A few anechoic pseudocysts, measuring between 0.2 and 0.3 cm, consistent with residual hyaluronic acid (HA) deposits (Fig 2, *A* and *B*).

The patient reported lip-filler injection about 1 year earlier in Azerbaijan, performed by a nonmedical professional. She could not specify the injected product or quantity but believed it was HA. Based on this history and on the ultrasound images, 450 IU of hyaluronidase was injected under ultrasound guidance into the affected regions, but no clinical improvement was observed.

**Fig 3:**
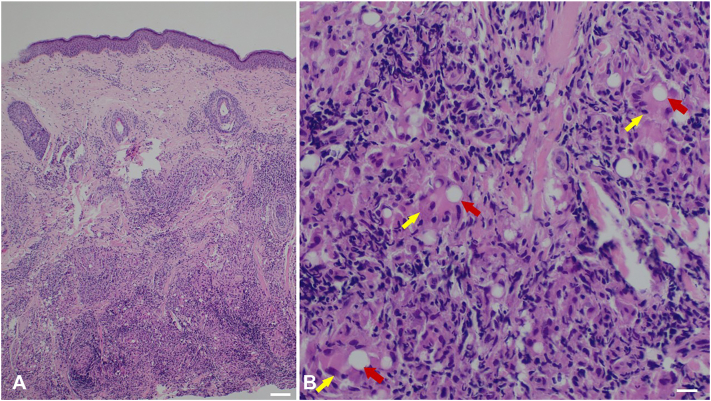
Histology of a skin biopsy reveals a heavy, deep reaching granulomatous inflammatory infiltrate **(A)** with admixed multinucleated histiocytic giant cells of the foreign body type (*yellow arrows*) **(B)** with characteristic intracellular PMMA microspheres (*red arrows*). (Hematoxylin and eosin staining, **(A)** 40× magnification, *white bar* is 200 μm, **(B)** 200× magnification, *white bar* is 50 μm). *PMMA*, Polymethyl methacrylate.

Given the lack of response after the first hyaluronidase session and the uncertainty regarding the injected material, a skin biopsy was performed for diagnostic clarification. While awaiting histologic results, and based on the ultrasonographic appearance showing both hyperechoic PMMA-like foci and anechoic nodules suggestive of residual HA, a second injection of hyaluronidase (600 IU) combined with triamcinolone acetonide (20 mg/mL) was attempted, resulting in only mild improvement. Histopathology subsequently revealed a deep foreign-body granulomatous reaction with characteristic PMMA microspheres and no evidence of HA (Fig 3).

**Fig 4:**
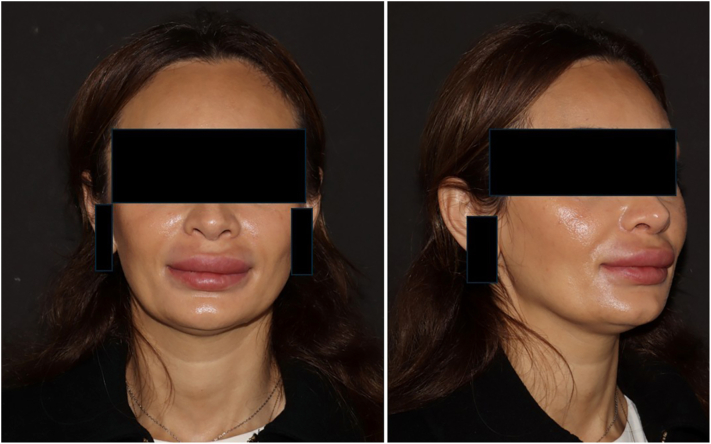
Patient 8 weeks after starting oral methotrexate (10 mg/week) and 4 weeks after the last intralesional triamcinolone injection, showing visible reduction of lip edema and perioral livedo.

After normal laboratory results, systemic therapy with oral methotrexate (10 mg/week) and folic acid (5 mg/week) was initiated. Three weeks after starting methotrexate, she showed visible reduction of upper lip edema and erythema. Another intralesional injection of triamcinolone (20 mg/mL) was performed while maintaining oral methotrexate. After 8 weeks of therapy, the patient demonstrated marked improvement, with a significant regression of edema and overall satisfaction with the outcome (Fig 4). She remains under regular clinical and laboratory follow-up with good tolerance to treatment.

## Discussion

The presence of nonbiodegradable PMMA microspheres within the tissues may, over time, elicit a foreign-body–type granulomatous inflammatory reaction. Because of their smooth surface and diameter between 30 and 50 μm, these particles cannot be phagocytosed and remain encapsulated by macrophages and multinucleated giant cells. This low-grade inflammatory response may remain quiescent for years and be reactivated by various immunologic triggers.^6^^,^^7^

Histologically, PMMA microspheres are surrounded by a chronic inflammatory infiltrate composed of lymphocytes, histiocytes, and multinucleated giant cells, along with peripheral fibrotic tissue. As these elements become confluent and closer together, dense nodular tissue forms, corresponding clinically to the palpable indurated nodules observed in symptomatic cases.^2^^,^^3^

Although surgical excision remains the gold standard, it is a technically challenging procedure associated with a risk of residual deformity and therefore reserved for refractory cases.^3^^,^^4^ Methotrexate, by inhibiting lymphocyte proliferation and suppressing the production of proinflammatory cytokines (such as tumor necrosis factor-α and interleukin-1), has emerged as a promising therapeutic alternative for delayed granulomatous reactions induced by PMMA.^5^^,^^6^ Alternative therapeutic options such as intralesional laser therapy (diode or Nd:YAG) have also shown promising results, although controlled studies are still lacking.^3^^,^^6^

Beyond physical symptoms, PMMA-related reactions can profoundly affect patients’ quality of life. The Berlin Injectable Filler Safety study demonstrated that individuals treated with permanent fillers had significantly higher mean Dermatology Life Quality Index scores than those who received biodegradable materials, reflecting psychosocial impairment comparable to that observed in chronic inflammatory dermatoses such as psoriasis and atopic dermatitis.^8^

An important challenge in this case, as well as in others managed clinically with immunosuppressive therapy, is determining the appropriate duration of treatment. Because the implant was not removed, discontinuation of therapy may be difficult and could trigger recurrence of symptoms. Nevertheless, nonsurgical management may be indicated in situations where removal of the material is anatomically challenging and likely to result in postoperative deformities or sequelae.

## Conclusion

This case highlights methotrexate as a promising nonsurgical therapeutic option for PMMA-induced granulomatous reactions, demonstrating favorable clinical improvement without the need for surgical excision. Accurate histologic confirmation remains essential for identifying the injected material in patients presenting with delayed inflammatory nodules or delayed filler reactions of uncertain composition, as proper diagnosis directly guides management. Further studies with larger number of participants and long-term follow-up are needed to better define standardized treatment protocols and to clarify the long-term efficacy and safety of pharmacologic approaches in PMMA-related inflammatory reactions.

## Conflicts of interest

None disclosed.
